# Global Emergence of Trimethoprim/Sulfamethoxazole Resistance in *Stenotrophomonas maltophilia* Mediated by Acquisition of *sul* Genes

**DOI:** 10.3201/eid1304.061378

**Published:** 2007-04

**Authors:** Mark A. Toleman, Peter M. Bennett, David M.C. Bennett, Ronald N. Jones, Timothy R. Walsh

**Affiliations:** * School of Medicine at Cardiff University, Cardiff, Wales; † The JONES Group/JMI Laboratories, North Liberty, Iowa, USA

**Keywords:** *Stenotrophomonas maltophilia*, *sul* genes, trimethoprim, integrons, IS*CR*, common regions, resistance, research

## Abstract

Trimethoprim/sulfamethoxazole (TMP/SMX) resistance remains a serious threat in the treatment of *Stenotrophomonas maltophilia* infections. We analyzed an international collection of 55 *S. maltophilia* TMP/SMX-sensitive (S) (n = 30) and -resistant (R) (n = 25) strains for integrons; *sul*1, *sul*2 and *dhfr* genes; and insertion element common region (IS*CR*) elements. *sul*1, as part of a class 1 integron, was detected in 17 of 25 TMP/SMX-R. Nine TMP/SMX-R strains carried *sul*2; 7 were on large plasmids. Five TMP/SMX-R isolates were positive for IS*CR2,* and 4 were linked to *sul*2; 2 others possessed IS*CR3*. Two IS*CR2*s were adjacent to *flo*R. Six TMP/SMX-S isolates harbored novel IS*CR* elements, IS*CR9* and IS*CR10.* Linkage of IS*CR3*, IS*CR9,* and IS*CR10* to *sul*2 and *dhfr* genes was not demonstrated. The data from this study indicate that class 1 integrons and IS*CR* elements linked to *sul*2 genes can mediate TMP/SMX resistance in *S. maltophilia* and are geographically widespread, findings that reinforce the need for ongoing resistance surveillance.

## Abstract 2

The first *sul*2 genes have been found in *S. maltophilia* from several different countries.

Nosocomial *Stenotrophomonas maltophilia* are intrinsically resistant to a plethora of antimicrobial agents that severely limit commonly used empiric standard antimicrobial therapies. *S. maltophilia* is resistant to many β-lactams, β-lactamase inhibitors, and aminoglycosides (*1**,**2*). A recent survey of SENTRY (www.jmilabs.com) Antimicrobial Surveillance Program isolates indicated that the newer fluoroquinolones demonstrated good efficacy; the most active were levofloxacin (6.5% resistance) and gatifloxacin (14.1%) (*3*). Furthermore, the resistance to the polymixins (20%–32%) is higher than observed in *Pseudomonas aeruginosa* (*3*,*4*). Because of low resistance levels (≈5%), trimethoprim/sulfamethoxazole (TMP/SMX) remains the therapy of choice worldwide. A recent study encompassing data from Europe, Latin America, and North America indicates that the level of resistance to TMP/SMX is 3.8%; however, previous studies indicate that the level is higher in Latin America than North America (*5**,**6*). Although surveillance studies are few, resistance to TMP/SMX appears to be emerging, and recent in vitro modeling studies have shown that combination therapies of TMP/SMX plus ciprofloxacin and TMP/SMX plus tobramycin exhibit a greater killing capacity then TMP/SMX alone (*7*,*8*).

*S. maltophilia* exhibits an array of mechanisms that singularly or collectively contribute to its multidrug resistance status. Intrinsic resistance includes inducible efflux pumps (*2*) and multiple β-lactamase expression (*1*) but not mutations in the quinolone resistance–determining region (*9*). In addition, *S. maltophilia* can acquire resistance through integrons, transposons, and plasmids (*10*). Recently, class 1 integrons have been characterized from *S. maltophilia* strains isolated in Argentina and Taiwan, which indicates that they contribute to TMP/SMX resistance through the *sul*1 gene carried as part of the 3′ end of the class 1 integron (*10*).

In addition to class 1 integrons, other mobile elements are associated with *sul* genes. For example, *Vibrio cholerae* serogroup 0139 is resistant to several antimicrobial agents, including SMX, and it has been recently shown that the *sul*2 gene was part of a cluster located on a novel genetic element of the integrative conjugative element group named SXT. The resistance genes harbored by SXT are embedded in a composite transposon-like structure and were probably acquired recently (*11*). Within this antimicrobial drug resistance region, an insertion element common region (ISC*R*) sequence, IS*CR2,* is adjacent to a *sul*2 gene that moves by 1-ended transposition. Thus, the possibility exists that *sul*2 genes can transfer intra- and intergenerically, including into *S. maltophilia*. Herein, we describe the molecular characterization of an international collection of *S. maltophilia* isolates and determine their mechanism of resistance to TMP/SMX, including the first report of *sul*2 genes and the first description of insertion element common region (IS*CR*) elements carried in *S. maltophilia*.

## Methods and Materials

### Bacterial Strains

During 1998–2003, a total of 1,744 *S. maltophilia* isolates collected worldwide were forwarded to the SENTRY Program (Europe, USA, and Australia) and tested for antimicrobial drug susceptibility. A TMP/SMX resistance phenotype was demonstrated for 71. From these isolates, 25 nonclonal strains from patients in North America, Latin America, and Europe were analyzed by using molecular methods together with 30 representative isolates that were TMP/SMX-susceptible. Isolates were identified by using the Vitek System and confirmed by using API20NE (bioMérieux, Hazelwood, MO, USA).

### Susceptibility Methods

Isolates were tested for susceptibility to TMP/SMX according to procedures of the Clinical and Laboratory Standards Institute (CLSI, formerly the National Committee for Clinical Laboratory Standards [NCCLS]) (*12*,*13*) by using broth microdilution methods (TREK Diagnostics, Cleveland, OH, USA). MIC results were confirmed with TMP/SMX Etests was performed according to the manufacturer’s directions (AB Biodisk, Solna, Sweden).

### Molecular Materials

PCR primers were purchased from Sigma-Genosys Ltd. (Pampisford, UK) and are listed in the Table. General reagents for DNA manipulation were obtained from Invitrogen (Groningen, the Netherlands). All other reagents were obtained from Sigma Chemical Co. or BDH (both of Poole, England, UK).

**Table:** Oligonucleotide primers used in this study, Cardiff, 2007

Primer	Sequence (5′→3′)	GenBank accession no.	Target	Reference
Ina-F	GCCTGTTCGGTTCGTAAGCT		*intI*	(*14*)
Int-R	CGGATGTTGCGATTACTTCG		*intI*	(*14*)
sul1-F	ATGGTGACGGTGTTCGGCATTCTGA		*sul1*	(*15*)
sul1-R	CTAGGCATGATCTAACCCTCGGTCT		*sul1*	(*15*)
sul2-F	GAATAAATCGCTCATCATTTTCGG	AJ289135	*sul2*	(*15*)
sul2-R	CGAATTCTTGCGGTTTCTTTCAGC	AJ289135	*sul2*	(*15*)
aacA4-F	AACTTGCGAGCGATCCGATG		*aacA4*	(*14*)
aacA4-R	ATGTACACGGCTGGACCATC		*aacA4*	(*14*)
aacA7-F	AATGGATAGTTCGCCGCTCG		*aacA7*	This study
aacA7-R	TTCCGGAAGCAGCGTACTTG		*aacA7*	This study
CRF	CACTWCCACATGCTGTKKC	AF231986	All IS*CR*	This study
CRF-r	GMMACAGCATGTGGWAGTG	AF231986	All IS*CR*	This study
CRFF	GGRYGCAACGSCTCAAGCG	AF231986	All IS*CR*	This study
CRFF-r	CGCTTGAGSCGTTGCRYCC	AF231986	All IS*CR*	This study
LECR2	CACTGGCTGGCAATGTCTAG	AF231986	IS*CR2*	This study
RECR2	CTTTGGACCGCAGTTGACTC	AF231986	IS*CR2*	This study
FloF	TCGACATCCTGGCTTCACTG	AF231986	*floR*	This study
FloR	ATTACAAGCGCGACAGTGGC	AF231986	*floR*	This study
dfra20f	GGGAAACACCGAGAAATGGG	AJ605332	*dfrA20*	This study
dfrA20R	TTCTTCTTCCCATTCTCCCC	AJ605332	*dfrA20*	This study
dfrA9F	CAGATTCCGTGGCATGAACC	X57730	*dfrA9*	This study
dfrA9R	GACCTCAGATACGAGTTTCC	X57730	*dfrA9*	This study
dfrA10F	TGTAGCGCGTGGTGTAAACG	AY055428	*dhfr10*	This study
dfrA10R	ACGTCTACGTGAGTATCCGC	AY055428	*dhfr10*	This study
strAF	TCTGTCGCACCTGCTTGATC	AY055428	*strA*	This study
strAR	CATTGCTGATGAACTGCGCG	AY055428	*strA*	This study
tetAF	CGCTGTTTGTGATTACACCC	AJ250203	*tetA*	This study
tetAR	CAGCGAGATGCGATATATCC	AJ250203	*tetA*	This study
glmmR	GAGTCAACTGCGGTCCAAAC	AJ289135	*glmM*	This study
glmmF	ACGGTATTCGTGGCAAAGCC	AJ289135	*glmM*	This study

### Strain Typing

Clonality among the *S. maltophilia* isolates was assessed by pulsed-field gel electrophoresis (PFGE) followed by *Xba*I digestion of genomic DNA. This assessment was conducted according to the standard 1-day protocol (*16*).

### Plasmid Isolation

Bacterial plasmids were isolated by the alkaline lysis method described by Grinsted and Bennett (*17*). Essentially, an overnight 10-mL culture was centrifuged (12,000× *g*) and suspended in water (250 μL) before 200 μL of lysis solution (0.2 mol/L NaOH, 1% sodium dodecyl sulfate [SDS]) was added. After lysis, 125 μL of neutralizing solution (0.3 mol/L potassium acetate, 1 mmol/L EDTA) was added. After precipitation, the suspension was centrifuged (12,000× *g*) and washed twice with 500 μL of a 50/50 (v/v) phenol/chloroform solution. The DNA was precipitated from the solution with the addition of 0.7 volumes of iso-amyl alcohol. The DNA/RNA pellet was washed twice in 1 mL 70% ethanol before being dried. The DNA was dissolved in 30 μL with 0.1 U RNase.

### Southern Hybridization

IS*CR* and *sul*2 PCR product amplified with primers CRF/CRFF-r were labeled with P^32^-ATP by random primer extension by using a commercially available kit (Stratagene, Amsterdam, the Netherlands) according to the manufacturer’s instructions. Unincorporated nucleotides were removed by passing the labeled DNA through a Sephadex column (Nick column, Pharmacia Bio-tech, Uppsala, Sweden).

Agarose gels used for Southern transfer were denatured for 45 min in denaturing solution (0.5 mol/L NaOH, 1.5 mol/L NaCl) before being neutralized in 0.5 mol/L Tris-HCl, pH7.5, 1.5 mol/L NaCl for 30 min. DNA was then transferred to Hybond (Amersham, Buckinghamshire, England, UK) nylon membrane by vacuum by using a custom-made Southern blotting apparatus. The nylon filter was prehybridized for at least 2 h with a blocking solution (6× SSC [1× SSC is 0.15 mol/L NaCl plus 0.014 mol/L sodium citrate], 0.1% [w/v] polyvinylpyrrolidone 400, 0.1% Ficoll [v/v], 0.1% bovine serum albumin, 0.5% SDS, 150 μg/mL denatured calf thymus DNA) at 65°C. The labeled denatured probe was then added to the solution and incubated overnight at 65°C. Finally, the filter was washed (300 mL 2× SSC, 0.1% [w/v] SDS followed by 0.1× SSC 0.1% SDS) at 65°C. Autoradiographic images were recorded on Hyperfilm-MP (Pharmacia Bio-tech), which was exposed overnight with intensifying screens.

### PCR Analysis

The presence of class 1 integrons in each strain was assessed by using class 1 specific primers. Gene cassettes embedded within the class 1 integrons were determined by using primers listed in the Table. Isolates were also screened for *sul*1, *sul*2, and *sul*3 by using sul1-F and -R, sul2-F and -R, and sul3-F and -R, respectively. Seven positive class 1 integron PCR products were chosen randomly, extracted from agarose gels after size separation, and sequenced with IntF, IntR, and custom-made oligonucleotide primers (Table).

The presence of IS*CR* elements in each strain was also determined by using primers CRF/CRFF-r designed to amplify the same 700-bp fragment internal to the open reading frames (ORFS) of IS*CR1–5* (Table). Full-length IS*CR2* elements were amplified with primers designed to target the ends of IS*CR2*. Primers used to amplify genes often associated with IS*CR2* or IS*CR3* are also given (Table). Because *dhfr* genes are associated with IS*CR* elements, we also performed molecular analysis of them.

PCRs were conducted in a final volume of 20 μL by using 10 μL ABgene Expand Hi-fidelity Master Mix (ABgene House, Surrey, England, UK). Primers were used at final concentrations of 10 μmol/L, and 1 μL of an overnight bacterial culture (optical density 1.0 at 600 nm) was added as source of DNA template. The cycling parameters were as follows: 95°C for 5 min, followed by 30 cycles of 95°C for 1 min, 55°C for 1 min, and 68°C for 1–4 min, depending on the sequence to be amplified, and ending with a 5-min incubation at 68°C.

### DNA Sequencing and Analysis

Sequencing was conducted on both strands by the dideoxyl-chain termination method with a Perkin-Elmer Biosystems 377 DNA sequencer (Perkin-Elmer, Waltham, MA, USA). Sequence analysis was performed with the Lasergene DNASTAR software package (SelectScience Ltd., Bath, England, UK). Sequence alignments were conducted with the ClustalW program (www.ebi.ac.uk/clustalw) and the PAM 250 matrix.

The sequence of IS*CR2*, together with the adjacent *sul*2 region and the novel IS*CR9* and IS*CR10,* has been deposited in GenBank. The genetic locus IS*CR2*-*glmM*/*sul*2 from isolates 5232, 4647, 3800, and 2107 has been attributed the accession nos. AM182031, 182030, 182029, and 181666, respectively. IS*CR9* and IS*CR10* have been given the numbers AM182033 and AM182032, respectively.

## Results

### TMP/SMX MICs

TMP/SMX MICs separated the isolates into an obvious bimodal distribution. The TMP/SMX-resistant isolates possessed MICs >32 mg/L, whereas the sensitive controls used as molecular comparators possessed TMP/SMX MICs ranging from 0.5 to 2 mg/L (Appendix Table).

### Detection and Determination of Class 1 Integrons

**Figure 1:**
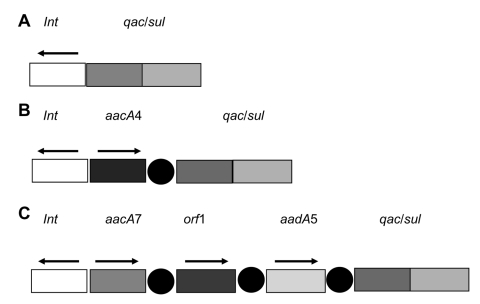
Schematic diagram of class 1 integrons from *Stenotrophomonas maltophilia* isolates. A), isolates 1893 and 9431. B) isolates 489, 9189, and 12221; C) isolates 3438 and 3444. Arrows depict direction of transcription, and shaded boxes represent gene cassettes found within the integron. The dark circles represent the 59-bp region immediately 5′ to the incorporated gene cassette.

Of the 25 TMP/SMX-resistant *S. maltophilia* isolates that we analyzed, 17 possessed the *sul*1 gene as part of the 3′ end of a class 1 integron. None of the TMP/SMX-susceptible *S. maltophilia* isolates yielded positive *sul*1 PCR products. PFGE analysis (data not shown) showed that only 2 isolates (9189 and 12221 from Chile) are clonally related (Appendix Table). To our knowledge, this is the first report of *sul*1-positive *S. maltophilia* isolates from North America and Europe. The *sul*1-positive isolates are widespread, being from Europe, North America, and South America. Most (5) were isolated from Brazil. The integrons associated with the *sul*1 gene vary in size; however, when 2 strains were isolated from the same country (e.g., 3438 and 3444, 9189 and 12221, and 98 and 14469), they possessed integrons of the same size, despite not being clonally related (Appendix Table). Seven of these integrons were randomly selected to examine their gene cassettes. The genetic context of the class 1 integrons and procured gene cassettes are shown in Figure 1. Strains 1893 (Germany) and 9431 (Brazil) possessed only the *int* and *sul*/*qac* genes. The class 1 integrons from strains 4891 (USA), 9189 (Chile), and 12221 (Chile) contained an embedded *aacA4* gene cassette. The 2 Mexican strains (3438 and 3444) contained 2 aminoglycoside-modifying genes (*aacA7* and *aadA5*) and an unknown ORF (Figure 1) yet were clonally unrelated, as judged by PFGE profiling. None of the integrons were the same as those characterized from strains isolated from Argentina (*10*).

### Detection and Location of *sul*2 Genes

All 55 isolates (both TMP/SMX resistant and sensitive) were screened for *sul*2 genes with the primers listed in the Appendix Table. Nine of the isolates gave PCR products for *sul*2. None of the TMP/SMX-susceptible *S. maltophilia* isolates displayed positive *sul*2 PCR products. Sequence analysis showed 100% identity with previous *sul*2 sequences.

Given that *sul*2 is normally located on medium-to-large sized plasmids, plasmids were isolated and characterized for *sul*2 carriage. Plasmid DNA was prepared from each isolate and used as a template for PCRs by using the *sul*2 primer detection set. In every case, a product of the size expected of *sul*2 sequence amplification was obtained. The purity of each plasmid preparation was evaluated by attempted PCR amplification of the host cell chromosomal *gyrA* gene. In no case was an amplification product obtained when plasmid DNA was used as template; in contrast, a *gyrA* amplification product of the correct size was obtained from genomic DNA. These data were later confirmed by Southern hybridization that used the labeled *sul*2 gene as a probe (data not shown). Unsurprisingly, in most cases *sul*2 was found on a large plasmid of ≈120 kb; however, in 2 of 9 *sul*2-positive isolates, *sul*2 gene was chromosomally encoded.

### Detection of IS*CR* Elements in TMP/SMX-sensitive and -resistant Strains

The s*ul*2 gene and *dhfr* genes are often found on plasmids and in close association with class 1 integrons or IS*CR* mobile genetic elements (*10*,*15**,**18**,**19*). Accordingly, we investigated the 55 *S. maltophilia* isolates for IS*CR* elements. Seven of the 25 TMP/SMX-resistant isolates yielded PCR products of the expected size (≈700 bp) when the IS*CR* specific primers CRF/CRFF-r were used, and 6 of 23 TMP/SMX-sensitive *S. maltophilia* isolates also yielded the correct-sized amplification products.

To determine whether the locations of the IS*CR* sequences in the *S. maltophilia* isolates are chromosomal or plasmid mediated, plasmid DNA was prepared from each isolate and used as a template for IS*CR*-PCR and Southern hybridization analysis in a similar manner as described for *sul*2. In every case, a product of the size expected of IS*CR* sequence amplification was obtained. Hence, in those isolates that possess an IS*CR* element, the element is located on a plasmid (data not shown). The PCR IS*CR* amplification products were recovered, purified, and ligated into the cloning vector, PCR-Topo-2.1 (Invitrogen) and recombinant plasmids were recovered by transformation of *Escherichia coli* DH5α. One clone from each transformation was chosen for further study.

Sequence analysis showed that 5/7 amplicons obtained from TMP/SMX-resistant *S. maltophilia* isolates were identical to the equivalent sequence of IS*CR2;* the other 2 amplicons were identical to that of IS*CR3* (Appendix Table). IS*CR2* sequences were identified in isolates originating from North and South America, as well as from Europe. In contrast, the IS*CR3* sequence was identified only in isolates that originated from Spain.

The IS*CR-*like elements carried by the sensitive isolates, while clearly related to IS*CR1–5*, differed markedly from known IS*CR* sequences (*15*). Two variants were found, which we have designated IS*CR9* and IS*CR10*. The putative amino acid sequences of IS*CR9* and IS*CR10* are ≈95% identical to each other and display 30%, 48%, and 74% identity to IS*CR2*, IS*CR,3* and IS*CR5*, respectively (Appendix Figure). These novel IS*CR*s are harbored in isolates from several different regions, including South American countries, the United States, and Turkey (Table).

### Identification of Resistance Genes and Sequences Adjacent to IS*CR* Elements

**Figure 2:**
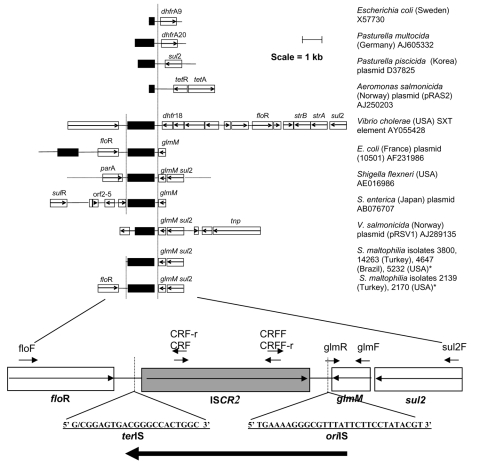
Genetic context of various insertion element common region (IS*CR2*) elements. Schematic of various GenBank sequences that harbor the IS*CR2* element. Open reading frames (ORFs) are depicted as open boxes with the direction of transcription of the various ORF indicated with arrows. The ORF of the IS*CR2* transposase is highlighted, and the limits of the IS*CR2* element are shown with vertical lines. The IS*CR2* element consists of an ORF of 1491 bp together with ≈120 bp of upstream sequence and ≈240 bp of downstream sequence. The IS*CR2* element identified in this study associated with the *flo*R resistance gene is amplified to show primer sites and positions of the putative *ori*IS and *ter*IS of the IS*CR2* element. The **bold arrow** indicates the direction of rolling circle replication of the IS*CR2* element. GenBank accession numbers of the various sequences are indicated. *P., Pasteurella*; *A., Aeromonas*; S. *enterica*, *Salmonella enterica*; *S.* *maltophilia*, *Stenotrophomonas maltophila.* *This study.

IS*CR2* is often associated with various antimicrobial resistance genes, not least, genes mediating TMP/SMX resistance (Figure 2) (*15*). These and other genes normally associated with IS*CR2* were therefore analyzed; these included *dhf*rA10, *dhf*rA9, *dhf*rA20, *flo*R, *tet*R, *str*A, *sul*2, and *glmM* encoding a truncated phosphoglucosamine mutase. Pairs of oligonucleotides were used (Table) to genetically characterize all those *S. maltophilia* isolates that possessed an IS*CR* element.

The *flo*R gene was detected in isolates 2139 and 2170 (which also contains IS*CR2*) from Turkey and the United States, respectively, and in isolates 12044 and 12049 (which also contains IS*CR3*) from Spain. A truncated *glmM* allele (Δ*glmM*) was detected in all IS*CR2*-containing isolates, and *sul*2 was found in all IS*CR2*- and IS*CR3*-containing isolates. The *dhfr,* *tetR,* and *strA* genes were not detected.

Linkage of the IS*CR* element to Δ*glmM*, *sul*2, or *flo*R was then investigated by PCR analysis, i.e., the oligonucleotide pair CRFF/*sul*2F is expected to generate a product if the IS*CR* sequence is close to *sul*2 and downstream of it (Figure 2). Using this strategy, we found that IS*CR2* was linked to Δ*glmM* and *sul*2 in all isolates that possess IS*CR2*. The *flo*R gene was also found to be linked to IS*CR2*, on the opposite side from Δ*glmM* and *sul*2, in isolates 2139 and 2170 (Figure 2). Linkage of IS*CR3* to either *sul2* or *floR* was not demonstrated.

## Discussion

We report *ul*2 genes being present in *S. maltophilia* and contributing to TMP/SMX resistance. In most cases, *sul*2 was carried on large plasmids (≈120 kb), but as judged by Southern hybridization data, a few appear to be chromosomally encoded. This study also supports the findings of Barbolla et al. that *sul*1 present in *S. maltophilia* is associated with class 1 integrons (*10*). Herein, we have characterized *S. maltophilia sul*1 genes from North America, South America, Spain, Turkey, Italy, and Germany, and observed that all of them were associated with class 1 integrons.

Most studies of the location and dissemination of *sul*2 genes have concentrated on *Enterobacteriaceae*, such as *E. coli* and *Salmonella enterica*. A recent study by Antunes et al. found *sul*1, *sul*2, or *sul*3 genes in most Portuguese isolates (*18*); 24 of 200 isolates contained both *sul*1 and *sul*2. *sul*2 has also recently been identified in *S. enterica* from Brazil (*20*). Similar results have been reported from *E. coli* urinary tract isolates in which ≈26% of strains possessed both *sul*1 and *sul*2 genes (*21*). A biased study examining TMP/SMX-resistant *E. coli* recently reported that 15 of 20 isolates possessed *sul*2 and that 6 of those also carried *sul*1 on a class 1 integron (*14*). Additional studies of *E. coli* have shown the intercontinental predominance of *sul*1 through class 1 integrons (*22*). A study by Pei et al. demonstrated the correlation of anthropogenic activity with the presence of *sul* genes in environmental samples (*23*). However, none of the studies demonstrated the genetic origin of the *sul*2.

In addition to *sul* genes associated with plasmids and class 1 integrons, we investigated whether the *S. maltophilia* isolates possessed IS*CR* elements and whether these could be linked to *dhfr* or *sul* genes, as has been shown (*18*). Of the 25 TMP/SMX-resistant isolates, 6 harbored *sul*2 linked to IS*CR2*. However, we could not detect any *sul*3 genes. In the isolates with IS*CR2*, the element was directly linked to a deleted version of a phosphoglucosamine mutase gene, Δ*glm*M, as has been reported on other occasions (Figure 2). This arrangement is identical to those of 5 other sequences in the EMBL database, in *E. coli* isolated from cattle in France and Germany (*24*), in the plasmid pRVS1 isolated from a strain of *Vibrio salmonicida* from Norway, in a plasmid from a strain of *S. enterica* isolated in Japan, and on the chromosome of *Shigella flexneri* isolated in the United States (*18*,*24*). In all cases, Δ*glmM* and *sul*2 are linked to the end of IS*CR2* that accommodates the IS*91 ori*IS equivalent (Figure 2). The dual arrangement of Δ*glm*M and *sul*2 is also found in plasmids of marine psychotrophic bacteria isolated in Norway (GenBank accession no. AJ306553/4), but in these cases the IS*CR2* element appears not to be present.

Two of the isolates harbored a copy of the *flo*R gene immediately upstream of a copy of IS*CR2* (Figure 2), an arrangement identical to that reported on plasmids found in isolates of *E. coli* from cattle in France and Germany (*24*). The *S. maltophilia* isolates investigated in this study came from Turkey and the United States. Two isolates from Spain also carry the *flo*R gene but not IS*CR2*. Instead, the isolates possess copies of IS*CR3,* which do not appear to be linked to *flo*R. The finding of florfenicol-resistant traits on plasmids in different bacterial species from different countries highlights the wide geographic spread of this resistance mechanism. The location of *flo*R next to IS*CR2* is such that it is possible, if not probable, that the resistance gene can be cotransposed with the IS*CR* element.

The findings within this study are important for several reasons. First, this is, to our knowledge, the first report of IS*CR* elements being found in *S. maltophilia* isolates. In 6 cases, these were linked to *sul*2 genes responsible for the TMP/SMX-resistant phenotype. Moreover, these isolates were unrelated strains found in different countries. Second, since TMP/SMX is the mainstay therapy for *S. maltophilia* infections, the mobilization of *sul* genes by means of class 1 integrons and IS*CR* elements is likely to increase with TMP/SMX consumption. Third, most *sul*2 genes in this study have been found on plasmids, and *sul*2-containing plasmids can potentially confer an increase in bacterial “fitness” (*25*). As yet, such phenomena have only been explored in *Enterobacteriaceae*, and it has yet to be established whether *sul*2-carrying plasmids have such an additive effect in *S. maltophilia* or for that matter, other nonfermenting gram-negative bacilli.

These data suggest that microbiology laboratories need to carefully monitor *S. maltophilia* TMP/SMX resistance, which has the potential to increase by means of mobile elements. We also advocate the continued international surveillance of antimicrobial drug resistance that may act as early warning systems for this kind of resistance. Furthermore, yearly monitoring with molecular probes is advisable.

## Supplementary Material

Appendix Table Origin, TMP/SMX susceptibility profiles, and genetic characterization of sul and ISCR elements in Stenotrophomonas maltophilia isolates*

Appendix Figure Amino acid sequence alignment of the central regions from the novel insertion element common region (ISCR) elements, ISCR9 and ISCR10. These sequences are aligned with ISCR2 and ISCR3, also found within this study, and ISCR. A consensus sequence is provided in the line above each alignment and numbering reading left to right.
