# Supplementary material for: Global Emergence of Trimethoprim/Sulfamethoxazole Resistance in Stenotrophomonas maltophilia Mediated by Acquisition of sul Genes
Source: Emerg Infect Dis. 2007 Apr;13(4):559–65. doi: 10.3201/eid1304.061378 (PMC2725981; doi:10.3201/eid1304.061378)
Supplement: Appendix Table — Origin, TMP/SMX susceptibility profiles, and genetic characterization of sul and ISCR elements in Stenotrophomonas maltophilia isolates* [file 06-1378_appT-s1.pdf]

Appendix Table. Origin, TMP/SMX susceptibility profiles, and genetic characterization of *sul* and IS*CR* elements in *Stenotrophomonas maltophilia* isolates*

SENTRY isolate	Country	TMP/SMX MIC Etest	Integron size (kb)	*sul*1	*sul*2	Plasmid	Plasmid-mediated *sul*2	IS*CR*
7666	Argentina	>32	4.5	+	–	–	–	–
40–9046	Argentina	<0.5	–	–	–	–	–	–
40–53I	Argentina	<0.5	–	–	–	–	–	–
6147	Brazil	2	–	–	–	–	–	–
98	Brazil	>32	1.0	+	–	–	–	–
3932	Brazil	>32	4.5	+	+	+	+	–
4647	Brazil	>32	–	–	+	+	+	–
9431	Brazil	>32	0.4	+	–	–	–	–
12357	Brazil	>32	1.2, 1.5	+	–	–	–	–
14469	Brazil	>32	1.0	+	+	+	+	–
48–1528	Brazil	<0.5	–	–	–	–	–	–
48–115	Brazil	<0.5	–	–	–	–	–	IS*CR9*
48–1528	Brazil	<0.5	–	–	–	–	–	–
57–1526	Brazil	<0.5	–	–	–	–	–	–
57–303	Brazil	<0.5	–	–	–	–	–	–
12221	Chile	>32	1.0	+	–	–	–	–
9189	Chile	>32	1.0	+	–	+	–	–
43–2380	Chile	<0.5	–	–	–	–	–	IS*CR9*
43–2092	Chile	<0.5	–	–	–	–	–	–
43–197I	Chile	<0.5	–	–	–	–	–	–
3438	Mexico	>32	3.0	+	–	–	–	–
3444	Mexico	>32	3.0	+	–	–	–	–
45–11382	Mexico	<0.5	–	–	–	–	–	–
45–6651	Mexico	<0.5	–	–	–	–	–	–
7618	Venezuela	>32	4.0	+	–	–	–	–
49–6147	Venezuela	2	–	–	–	–	–	–
49–1036	Venezuela	<0.5	–	–	–	–	–	IS*CR10*
49–3886	Venezuela	<0.5	–	–	–	–	–	–
345	Canada	>32	1.4	+	–	–	–	–
38–1086	Canada	<0.5	–	–	–	–	–	–
38–3673	Canada	<0.5	–	–	–	–	–	–
867	USA	>32	–	–	–	+	–	–
489I	USA	>32	1.0	+	–	+	–	–
2170	USA	>32	–	–	+	+	+	IS*CR2*
4568	USA	2	–	–	–	–	–	–
1246	USA	1	–	–	–	–	–	–
1113	USA	1	–	–	–	–	–	–
1696	USA	1	–	–	–	–	–	–
15–6279	USA	<0.5	–	–	–	–	–	–
15–6290	USA	<0.5	–	–	–	–	–	IS*CR9*
4225	Belgium	>32	–	–	–	–	–	–
11131	France	1	–	–	–	–	–	–
12876	France	>32	–	–	+	+	+	–
1893	Germany	>32	0.4, 0.9	+	–	–	–	–
5232	Germany	>32			+	+	+	IS*CR2*
2597	Italy	>32	1.3, 1,5	+	–	–	–	–
75–12113	Italy	<0.5	–	–	–	–	–	–
75–16066	Italy	<0.5	–	–	–	–	–	–
12044	Spain	>32	–	–	+	+	–	IS*CR3*
12049	Spain	>32	0.4, 3.0	+	–	–	–	IS*CR3*
66–12258	Spain	<0.5	–	–	–	–	–	–
2139	Turkey	>32	–	–	+	+	–	IS*CR2*
3800	Turkey	>32	–	–	+	+	+	IS*CR2*
14263	Turkey	>32	1.0	+	–	–	–	IS*CR2*
69–2333	Turkey	<0.5	–	–	–	–	–	IS*CR10*
69–4959	Turkey	<0.5	–	–	–	–	–	IS*CR10*
