# Supplementary figures and images for: Global Emergence of Trimethoprim/Sulfamethoxazole Resistance in Stenotrophomonas maltophilia Mediated by Acquisition of sul Genes
Source: Emerg Infect Dis. 2007 Apr;13(4):559–65. doi: 10.3201/eid1304.061378 (PMC2725981; doi:10.3201/eid1304.061378)

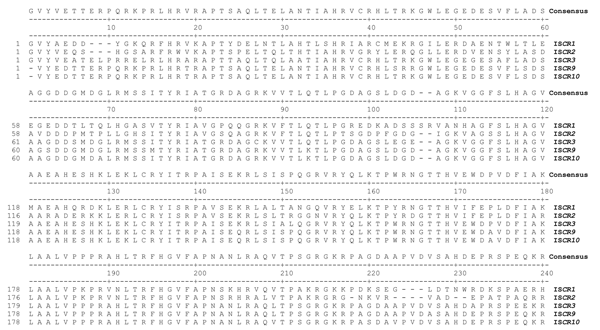

Supplement: Appendix Figure — Amino acid sequence alignment of the central regions from the novel insertion element common region (ISCR) elements, ISCR9 and ISCR10. These sequences are aligned with ISCR2 and ISCR3, also found within this study, and ISCR. A consensus sequence is provided in the line above each alignment and numbering reading left to right. [file 06-1378_appF-s1.gif]
